# Mental distress and nutrition of family physicians, a European based cross-sectional study

**DOI:** 10.1097/MD.0000000000039544

**Published:** 2024-09-13

**Authors:** Ozden Gokdemir, Genco Gorgu, Marina Jotić Ivanović, Angharad (Kate) Woolley, Ahmet Öztürk, Miriam Rey Seoane, Lukasz Reczek, Maria Bakola, Olgu Aygun, Halime Seda Küçükerdem, Hilal Toplu

**Affiliations:** a Izmir University of Economics, Faculty of Medicine, Izmir, Turkey; b Marmara District State Hospital/Turkey EYFDM – European Young Family Doctors Movement Mental Health SIG Lead; c Family Medicine Department, Primary Health Care Center Doboj, Doboj, Bosnia and Herzegovina EYFDM – European Young Family Doctors Movement Lifestyle Medicine SIG Lead; d University of Leicester, UK; e Bandirma District Health Directorate, Turkey; f ICS- Institud Catalá.de la Salut / CUAP Manso / Barcelona / Spain EYFDM – European Young Family Doctors Movement Emergency Medicine Lead; g College of Family Physicians, Warsaw, Poland; h Department of Public Health, University of Patras, Patras, Greece; i Department of Family Medicine, Bozyaka Research and Training Hospital, İzmir, Turkey; j Kadikoy Number 9 Health Center, Istanbul, Turkey.

**Keywords:** family physician, lifestyle medicine, mental distress, nutrition

## Abstract

Family physicians are a pillar of the primary healthcare system, and their own mental well-being is integral to their performance. However, many studies have suggested a high prevalence of mental distress. The contributing factors include the emotional demands of the profession, work overload, budgetary constraints, loss of autonomy, and erosion of professional values. Outbreaks such as the COVID-19 pandemic exacerbate distress due to a greater risk of exposure to the virus, increased working hours, and fear of infecting families. Thus, it is crucial to assess risks and provide preventive measures. This study aimed to evaluate the association between the dietary patterns of family physicians and their mood. This study used a cross-sectional descriptive method and a validated Food-Mood Questionnaire (FMQ), shared via social networks across 10 European countries, to collect data from family physicians. Permission to use the FMQ was obtained. The breakfast-pattern subscale had the highest mean score (14.670 ± 4.305). The other subscale mean scores were as follows: health pattern (13.317 ± 5.388), mental distress pattern (11.184 ± 3.824), and western diet pattern (9.827 ± 3.604). According to Pearson correlation test there was a positive correlation between breakfast and Western diet patterns and between breakfast and health patterns. There was a negative correlation between health and mental distress pattern. Evidence suggests that mental distress may arise from different dietary deficiencies. Physicians’ nutritional patterns have an impact on health indicators and are distributed in relation to sociodemographic factors, especially the regions they live in. Diet assessment is becoming a vital modifiable risk factor for mental health, but further research in this field is needed.

## 1. Introduction

The recent COVID-19 pandemic has negatively affected the well-being of healthcare professionals in several ways. Doctors and nurses in primary care settings are at high risk of COVID-19 transmission.^[1,2]^ Increasing working hours, ensuring patient and employee safety, and always being at the forefront impose a significant mental burden on employees, resulting in sleep, depression, and anxiety disorders.^[3–5]^ Healthcare professionals worldwide report working without rest, even without sufficient safety precautions.^[6,7]^ The pandemic has seen stigmatization and violence against healthcare professionals,^[8]^ as well as the lack of competent regulations on these issues,^[9]^ which have tested the resilience of medical staff.

Resilience is the ability of individuals to find strength to persevere in difficult circumstances,^[10]^ improving the sustainability of their practice. According to Sisto et al, characteristics supporting resilience are the ability to evaluate the situation, make decisions, cope with difficulties in a positive way, recover when faced with difficulties, use character strengths to overcome difficulties, provide mental calmness after the difficulty disappears, use personal development experiences, and positively use skills learned from dealing with previous difficulties in responding constructively to future challenges.^[11]^ Changes in “eating behavior” could be a coping mechanism.^[12]^

In 1989, a new approach, “lifestyle medicine,” entered medicine in a symposium on cancer.^[13]^ While lifestyle modifications can improve health status, many guidelines and institutions have also brought lifestyle medicine to the forefront.^[14–16]^ There has been a growing interest in the use of diet to improve mental well-being in patients.^[17]^ Our study aims to further the knowledge in this field by investigating the link between lifestyle practices (specifically diet) and mood and resilience in healthcare workers.

## 2. Methods

The research was designed as a multicenter, cross-sectional-analytical survey and was carried out between March 01, 2022, and June 06, 2022, with 249 participants from 8 countries. The sample size was determined using the OpneEpi program, with a confidence level of 95%. A sociodemographic data form and Food-Mood Questionnaire (FMQ) were used to design the data collection tool.

The questionnaire was used to create an Internet-based data form on Google Docs, which was shared via social networks via email, social media, and WhatsApp platforms to family physician groups across 10 European countries to collect data from family doctors between May 2022 and June 2022.

### 2.1 Materials

1. Demographic Information of Participants: Researchers created this part by reviewing the relevant literature and included questions related to demographic data, general health perception, habits, nutrition and physical activity status, and the practice of lifestyle medicine (LSM) in clinical practice.
2. The Food-Mood Scale: Begdache et al’s scale was administered in English. For construct validity, principal component analysis consists of 5 subscales: internal consistency (Cronbach alpha > 0.70) and internal reliability (intraclass correlation coefficient ranges between 0.619) and 0.884; *P* < .01 (confidence interval of 95%). This scale can be used as an approved and reliable tool with many potential applications that can be used prophylactically and therapeutically.^[18]^

Food Mood Questionnaire (FMQ) was developed by Begdache et al in 2019 to satisfy the need for short and validated questionnaires to evaluate dietary patterns and mental distress in clinical and research settings.^[18]^

Principal component analysis for construct validity generated 5 subscales that reflected internal consistency (Cronbach α > 0.70) and reliability (intraclass correlation coefficient ranged between 0.619 and 0.884; *P* < .01; confidence interval 95%). Further details of the development and validity testing of the questionnaire have already been published.^[18]^

The original questionnaire had twenty-one questions on a 6-point Likert scale (ranging from 0 = none of the time to 6 = more than 4 times). The original questionnaire consists of 5 dimensions. During the development of the FMQ, a component analysis was conducted within the scope of validity studies. As a result, the scale items were distributed under 5 patterns: mental distress, breakfast pattern, healthy pattern, Western diet pattern, and supplement pattern. The scale is additive; therefore, the total score is used.

### 2.2 Analyses

Exploratory analysis was performed using SPSS 22. When we applied the normality tests, we observed that the skewness and kurtosis of the dataset were between −1 and 1.^[19]^ The sample data were drawn from a normally distributed population within some tolerance; therefore, we decided to apply parametric tests to analyze the data.

For the analysis of the data set, the relationship between subscale scores and demographic characteristics was analyzed by 1-way ANOVA (independent *t* test for gender variables only), and the relationship between subscale scores was analyzed using the Pearson correlation test. post hoc analyses were applied to evaluate the variables that provided statistically significant differences in the results of the 1-way ANOVA test.

The study was approved by the Ethical Committee of Izmir University of Economics. Informed consent was obtained online at the beginning of the survey.

## 3. Results

### 3.1 Demographics

Participants were 249 family physicians. Of the participants, 197 (79.1%) were women, and 52 (20.9%) were men. Sociodemographic characteristics of the participants are presented in Table 1. A total of 249 physicians who answered our online survey were included in the study; 79.1% of the 249 participants were women and 55.8% were family medicine specialists. (Table 1).

**Table 1:** Frequency distribution of demographic variables

		N	%
Education	MSc/PhD	107	42.97
	University	142	57.03
Country	Bosna	30	12.0
	Greece	33	13.3
	Poland	53	21.3
	West Mediterranean	34	13.7
	East Mediterranean	38	15.3
	England	33	13.3
	Other	28	11.2
Age	24 to 30	53	21.3
	31 to 40	131	52.6
	41 to 50	39	15.7
	51 to 60	19	7.6
	61 and more	7	2.8
Gender	Female	197	79.1
	Male	52	20.9
Profession	FM specialist	139	55.8
	FM trainee	90	36.1
	Other	20	8.0
All		249	100

### 3.2 Explanatory Analysis

The breakfast-pattern subscale had the highest mean score (14.670 ± 4.305). The other subscale mean scores were as follows: health pattern (13.317 ± 5.388), mental distress pattern (11.184 ± 3.824), and western diet pattern (9.827 ± 3.604) (Table 2). When the participants’ health pattern points according to demographic variables were examined, the country with the highest mean score was Greece (18.7273, *P* = .01), the age group was 61 and more (15.4286, 0.015), and the professionals were family medicine specialists (14.0216, *P* = .047) (Table 3).

**Table 2:** Average points of subscales (standard deviation).

	Mean	Median	SD
Health pattern	13.317	13.00	5.388
Breakfast pattern	14.670	15.00	4.305
Western diet pattern	9.827	10.0	3.604
Mental distress pattern	11.184	11.00	3.824

**Table 3:** One-way ANOVA test is explaining association between health pattern and demographics.

Health pattern		N	Mean	Std. Deviation	*P*
Education	MSc/PhD	107	14.037	5.19	.333
	University	142	12.791	5.5	
Country	Bosna	30	12.5667	5.05612	**.001**
	Greece	33	18.7273	3.65951	
	Poland	53	11.1509	4.90446	
	West Mediterranean	34	14.9706	5.08405	
	East Mediterranean	38	11.1053	4.92533	
	England	33	13.9697	5.27663	
	Other	28	12.0714	4.59411	
Age	24 to 30	53	12,9811	5.51394	**.015**
	31 to 40	131	12.4733	5.34261	
	41 to 50	39	15.2564	5.29507	
	51 to 60	19	15.3158	4.17735	
	61 and more	7	15.4286	5.41163	
Gender	Female	197	13.2183	5.25066	.574
	Male	52	13.692	5.91952	
Profession	FM specialist	139	14.0216	4.93651	**.047**
	FM trainee	90	12.2222	5.66237	
	MD	20	13.3500	6.51537	

*Independent *t* test for sex variables.

When the relationship between breakfast-pattern score and various demographic variables was examined, it was observed that the country variable had a significant effect on breakfast-pattern score. Poland had the highest mean score (16.0189, *P* = .008) (Table 4).

**Table 4:** Breakfast-pattern score and demographic variables.

Breakfast pattern		N	Mean	Std. Deviation	p
Education	MSc/PhD	107	14.5377	4.32088	.104
	University	142	14.8849	4.16521	
Country	Bosna	30	14.9000	4.00302	**.008**
	Greece	33	15.4848	3.86589	
	Poland	53	16.0189	3.39961	
	West Mediterranean	34	15.2941	4.60256	
	East Mediterranean	38	13.6053	4.06394	
	England	33	13.7576	4.15354	
	Other	28	12.,6786	5.69635	
Age	24 to 30	53	14.5472	4.57235	.684
	31 to 40	131	14.6260	4.08814	
	41 to 50	39	14.7692	4.55066	
	51 to 60	19	15.7368	4.82864	
	61 and more	7	13.0000	3.82971	
Gender	Female	197	14.6701	4.22188	.996
	Male	52	14.6731	4.65146	
Profession	FM specialist	139	14.8705	4.45401	.314
	FM trainee	90	14.6667	3.92629	
	MD	20	13.3000	4.83518	

The mental distress score according to demographic data was analyzed with the 1-way ANOVA test; the country variable was found to be significant and Eastern Mediterranean countries had the highest mental distress pattern score (14.0526, *P* = 001). When we examined the relationship between the mental distress pattern mean score and the gender variable of the participants using the independent *t* test, the mental distress pattern subscale mean score of female physicians was 11.5736 and the mean score of male physicians was 9.7115 (*P* = .002) (Table 5).

**Table 5:** One-way ANOVA test is applied to show relationship between mental distress pattern subscale score and demographics.

Mental distress pattern		N	Mean	Std. Deviation	*P*
Education	MSc/PhD	107	14.37	5.19	.422
	University	142	12.791	5.55	
Country	Bosna	30	11.0333	3.65290	**.001**
	Greece	33	8.6061	3.92858	
	Poland	53	11.8491	3.12186	
	West Mediterranean	34	11.6765	3.71500	
	East Mediterranean	38	14.0526	3.66833	
	England	33	9.3636	2.88117	
	Other	28	10.7857	3.56274	
Age	24 to 30	53	11.7170	3.74873	.235
	31 to 40	131	10.8702	3.81172	
	41 to 50	39	11.6154	4.20911	
	51 to 60	19	11.8421	3.33772	
	61 and more	7	8.8571	2.96808	
Gender	Female	197	11,5736	3.75403	**.002**
	Male	52	9.7115	3.76431	
Profession	FM specialist	139	10.9065	3.68878	.127
	FM trainee	90	11.2667	3.72299	
	MD	20	12.7500	4.89764	

*Independent *t* test for gender variable.

We evaluated our independent variables that made a significant difference as a result of explanatory analyses using post hoc (Gabriel) analyses. post hoc (Gabriel) analysis showed statistically significant relationship between country and health pattern subscale scores. Post hoc (Gabriel) analysis showed an association between age and health pattern subscale scores. The difference that made the age variable significant was between the mean score differences of the 41 to 50 and 30 to 40 age groups. Another health pattern subscale mean score difference was found between family medicine specialists and family medicine trainees. The variables that showed striking results in terms of the breakfast-pattern mean score were the countries of the participants. The difference that makes the country variable significant is the mean breakfast score difference between Poland and other countries’ groups (Poland mean was higher). Another subscale in which the country variable was also significant in the post hoc analyses was the Western diet pattern. The mean score differences between Bosnia and Greece, Greece and East Mediterranean, England, Greece, and others. Greece mean score on the western diet was significantly higher than that of the East Mediterranean, Bosnia, and England.

Pearson correlation test was used to measure the relationship between the subscale scores. According to the analysis results, there was a positive correlation between breakfast and Western diet patterns and between breakfast and health patterns. There was a negative correlation between health and mental distress pattern.

## 4. Discussion

The observed negative correlation between health patterns and mental distress patterns may be mediated by functional constipation.^[20]^ While changes in “eating behavior “ could be 1 of the coping mechanisms,^[12]^ exercise is 1 of the positive coping strategies, not only for acute stress but also for healthy living.^[21]^

One of the results of our study revealed that Health (13.317) and Breakfast (14.670) subscales had higher mean scores. In another study, based on the statements of participating physicians and using a knowledge-based scale as in our study, Harkin et al found that 50% of physicians rated their personal diet as excellent or very good, whereas 13% rated their diets as fair or poor. Physicians most commonly reported that they recommended a Mediterranean diet (55.1%).^[22]^ In addition, The International Breakfast Research Initiative, which was launched as a multicenter study in 2018, emphasized the effect of breakfast on mood and its variability between cultures. There is evidence that breakfast habits support improved cognitive function, particularly short-term benefits on attention, executive function, and memory. One of the main motivations for the compilation of the relevant study and 1 of the results reflects that while similarities in energy intake at breakfast may exist across countries, there is also considerable diversity in the local choices of breakfast foods and in the cultural context of the breakfast meal itself. The timing and location of breakfast can vary widely across countries and population subgroups.^[23]^ According to the results of our study, sex was an independent variable with a significant statistical relationship with mental distress. The mental distress pattern score of the female participants was higher than that of the male participants. In a systematic evaluation study by Imamura et al, in which they evaluated diet quality by sex between 1987 and 2020 in 187 countries, healthy diet consumption scores of women were higher.^[24]^ The fact that the population in our sample was healthcare workers and that the data collection period coincided with the global coronavirus pandemic may have played a role in the emergence of these results. Women physicians, who were at the forefront of the fight against the disease, had to bear the weight of their gender roles and make a supreme sacrifice. This may have affected their nutritional behaviors.

One of the findings we obtained as a result of our study was the positive correlation between the participants’ western diet pattern score and breakfast-pattern scores. When we question the reasons for encountering a result contrary to the conventional literature data in the field of nutrition, it may be that the anxiety of grocery shopping is higher than the fear of contacting food, and this situation favors the Western diet pattern.^[25]^ One of the main results of our study was the negative correlation between mental stress and health pattern scores. In an exploratory qualitative study by Lemaire et al, a group of physicians, including primary care physicians, asked questions about the relationship between nutritional intake and cognitive function. Physicians report that inadequate workplace nutrition has a significant negative impact on their personal wellness and professional performance.^[26]^ Therefore, the results obtained by analyzing the quantitative data within the scope of our study support the preliminary data in the medical literature. The COVID-19 pandemic has affected eating behaviors at work and at home and has increased the rate of emotional eating. Hadar–Shoval study on the effects of unpredictable changes on eating behavior showed that poor emotional regulation poses health risks.^[27]^ Gender status and being affected by COVID-19 stressors were effective in improving sleep quality, alcohol consumption, cigarette smoking, physical activity, and emotional eating.

### 4.1 Limitations of the study

The data collection of the study was based on participants’ online statements, and because the participants were from various geographical regions and countries, it was necessary to choose between different meteorological views on the way of classification before the analysis.

## 5. Conclusion

In the present context, lifestyle medicine is crucial not only for patients grappling with non-communicable diseases but also for the well-being of primary care physicians actively involved in their prevention and management. The practice of medicine is a difficult profession, as it creates mental stress on individuals and affects their health indicators. This can be compounded by factors such as being a novice assistant physician or bearing the burden of gender discrimination as a female physician. Medical doctors serve as societal role models, and the awareness and attitudes of family physicians become particularly significant due to their holistic, patient-centered, and individual-engaged approach. The incorporation of lifestyle medicine practices among primary care physicians has the potential to enhance the overall management of non-communicable diseases. Eating a regular, balanced Mediterranean diet can improve the emotional state and health perception of physicians. However, the geographical location can influence dietary habits both biologically and culturally. There is a need for new research to clarify this influence and to organize evidence-based recommendations and health promotion campaigns. This improvement may manifest through elevated physician performance, contributing to improved well-being and serving as positive role models for patients. The open and deliberative stance of healthcare professionals plays a pivotal role in shaping patients’ health-seeking behaviors. Women physicians, who played a leading role in combating the disease, had to navigate the challenges associated with gender roles and made significant sacrifices. This may have impacted their dietary behaviors.

## Acknowledgments

All the authors thank Lina Begdache..

## Author contributions

**Conceptualization:** Ozden Gökdemir, Marina Jotić Ivanović, Olgu Aygun.

**Data curation:** Ozden Gökdemir, Genco Gorgu, Marina Jotić Ivanović, Angharad (Kate) Woolley, Miriam Rey Seoane, Lukasz Reczek, Maria Bakola, Halime Seda Küçükerdem, Hilal Toplu.

**Formal analysis:** Ozden Gökdemir, Ahmet Öztürk, Lukasz Reczek, Olgu Aygun, Halime Seda Küçükerdem, Hilal Toplu.

**Investigation:** Genco Gorgu, Marina Jotić Ivanović, Angharad (Kate) Woolley, Ahmet Öztürk, Miriam Rey Seoane, Maria Bakola.

**Methodology:** Ozden Gökdemir, Genco Gorgu, Angharad (Kate) Woolley, Ahmet Öztürk, Lukasz Reczek, Olgu Aygun, Hilal Toplu.

**Project administration:** Ozden Gökdemir.

**Resources:** Ozden Gökdemir, Genco Gorgu, Marina Jotić Ivanović, Angharad (Kate) Woolley, Miriam Rey Seoane, Maria Bakola, Halime Seda Küçükerdem, Hilal Toplu.

**Software:** Maria Bakola, Hilal Toplu.

**Supervision:** Ozden Gökdemir, Marina Jotić Ivanović, Ahmet Öztürk, Olgu Aygun.

**Validation:** Ozden Gökdemir, Genco Gorgu, Marina Jotić Ivanović, Angharad (Kate) Woolley, Lukasz Reczek, Olgu Aygun, Halime Seda Küçükerdem.

**Visualization:** Genco Gorgu, Angharad (Kate) Woolley, Ahmet Öztürk, Miriam Rey Seoane, Maria Bakola, Halime Seda Küçükerdem.

**Writing – original draft:** Ozden Gökdemir, Marina Jotić Ivanović, Angharad (Kate) Woolley, Ahmet Öztürk, Miriam Rey Seoane, Lukasz Reczek, Maria Bakola, Olgu Aygun, Halime Seda Küçükerdem, Hilal Toplu.

**Writing – review & editing:** Ozden Gökdemir, Marina Jotić Ivanović, Angharad (Kate) Woolley.
